# Comparison of gut microbiota in exclusively breast-fed and formula-fed babies: a study of 91 term infants

**DOI:** 10.1038/s41598-020-72635-x

**Published:** 2020-09-25

**Authors:** Jingran Ma, Zhenghong Li, Wenjuan Zhang, Chunli Zhang, Yuheng Zhang, Hua Mei, Na Zhuo, Hongyun Wang, Lin Wang, Dan Wu

**Affiliations:** 1 Department of Paediatrics, Peking Union Medical College Hospital, Chinese Academy of Medical Sciences, Beijing, 100730 China; 2 Department of Neonatology, Inner Mongolia People’s Hospital, Hohhot, 010010 China; 3 Department of Neonatology, The Affiliated Hospital of Inner Mongolia Medical University, Hohhot, 010050 China; 4 Department of Neonatology, Inner Mongolia Maternal and Child Health Hospital, Hohhot, 010020 China

**Keywords:** Microbial ecology, Paediatric research, Paediatrics, Gastrointestinal system

## Abstract

To compare gut microbiota of healthy infants that were exclusively breast-fed or formula-fed, we recruited 91 infants, who were assigned into three different groups and fed by breast milk (30 babies), formula A (30 babies) or formula B (31 babies) exclusively for more than 4 months after birth. Faecal bacterial composition was tested. Among different groups, α diversity was lower in breast-fed group than formula-fed groups in 40 days of age, but increased significantly in 6 months of age. The *Bifidobacterium* represented the most predominant genus and *Enterobacteriaceae* the second in all groups. In 40 days of age, *Bifidobacterium* and *Bacteroides* were significantly higher, while *Streptococcus* and *Enterococcus* were significantly lower in breast-fed group than they were in formula A-fed group. *Lachnospiraceae* was lower in breast-fed than formula B-fed group. *Veillonella* and *Clostridioides* were lower in breast-fed than formula-fed groups. In 3 months of age there were less *Lachnospiraceae* and *Clostridioides* in breast-fed group than formula-fed groups. There were also significant differences of microbiota between formula A-fed and formula B-fed groups. Those differences may have impacts on their long-term health.

## Introduction

The gut microbiota at birth is of low diversity, while a more complex composition is established by 1–2 years of age to be similar with gut microbiota of adults^1^. The first year of life is pivotal to the development of gut microbiota, with breast milk being the main influence factor to the composition of microbiota^2,3^.

Numerous data have shown an association between gut microbiota and chronic non-infectious diseases in humans. The development of gut microbiota in early life has impacts on later health^4^. The gut microbiota affects the immune system maturation, nutrient absorption, as well as avoids pathogen colonization. Changes in gut microbiota composition have associations with long-term health disorders, for example obesity, atopic diseases, and chronic inflammatory diseases. So there is a window of opportunity to regulate the gut microbiota in early life to promote long-term health^5^.

Human breast milk is an ideal source of nutrients for infants, which contains a large variety of components. Breast milk also influences health promoting microorganisms by factors such as polymeric IgA (pIgA), antibacterial peptides, and components of the innate immune response^6^. Compared with formulas, breast milk has superior effects on the barrier integrity and mucosal defences of the intestinal tract^1^. However, breast milk is not available in many circumstances. While the composition of commercial formulas is more and more close to that of breast milk, gut microbiota of breast-fed and formula-fed babies remains distinct^7^.

Studies of gut microbiota in babies fed exclusively breast milk or formulas are rare and mostly of small-scale. Actually, babies are partially breast-fed or formula-fed in most research articles. To gain a better understanding of how different feeding patterns affect the gut microbial composition, we conducted a study detecting gut microbiota in babies fed exclusively human milk or a certain kind of formulas for more than 4 months after birth. What’s more, in our study, solid foods were introduced from 4 to 6 months of age, so they did not affect the microbiota before 4 months of age, ruling out the impact of solid foods on microbiota.

## Results

### Basic characteristics

A total of 91 infants were enrolled finally (49 males; 42 female; male/female = 1.17; p value of inter group comparison = 0.177) with a mean gestational age of 39.3 ± 1.1 weeks (range, 37–42 weeks; p = 0.342), birth weight of 3316.9 ± 406.8 g (2500.0–4400.0 g; p = 0.136), the values of the 25th and 75th percentile (P25/P75) birth length of 49.0/51.0 cm (45.0–55.0 cm; p = 0.137), and birth head circumference of 33.7 ± 0.8 cm (32.0–35.0 cm; p = 0.895). The data mentioned above had no significant difference among three groups (Table 1).

**Table 1:** Basic characteristics at birth.

Group	Total number (n)	Male/female	Gestational age (week)	Delivery mode (ratio = VD/CS^a^)	Birth weight (g)	Birth length(cm)	Head circumference (cm)
Breast-fed	30	12/18	39.4 ± 1.1	1.1	3070.0/3545.0^a^	48.0/51.8^b^	33.8 ± 1.3
Formula A-fed	30	19/11	39.0 ± 1.1	0.6	2875.0/3512.5^a^	50.0/51.0^a^	33.7 ± 0.8
Formula B-fed	31	18/13	39.1 ± 1.1	0.7	3200.0/3700.0^a^	50.0/51.0^a^	33.6 ± 0.8
p value	–	0.177^c^	0.342^d^	0.663^c^	0.136^c^	0.137^c^	0.895^c^

^a^VD/CS: vaginal delivery/caesarean section delivery.

^b^The values of the 25th and 75th percentile (P25/P75).

^c^Inter-group differences were evaluated by χ^2^ test for categorical variables.

^d^Variables were statistically tested by Kruskal Wallis test.

Four cases dropped out, and finally there were 30 babies in breast-fed group (12 males; 18 female), 30 babies in formula A-fed group (19 males; 11 female) and 31 babies in formula B-fed group (18 males; 13 female) enrolled. Totally 81 stool samples in 40 days of age (40 days), 80 samples in 3 months of age (3 m) and 68 samples in 6 months of age (6 m) were collected.

### α diversity

**Figure 1:**
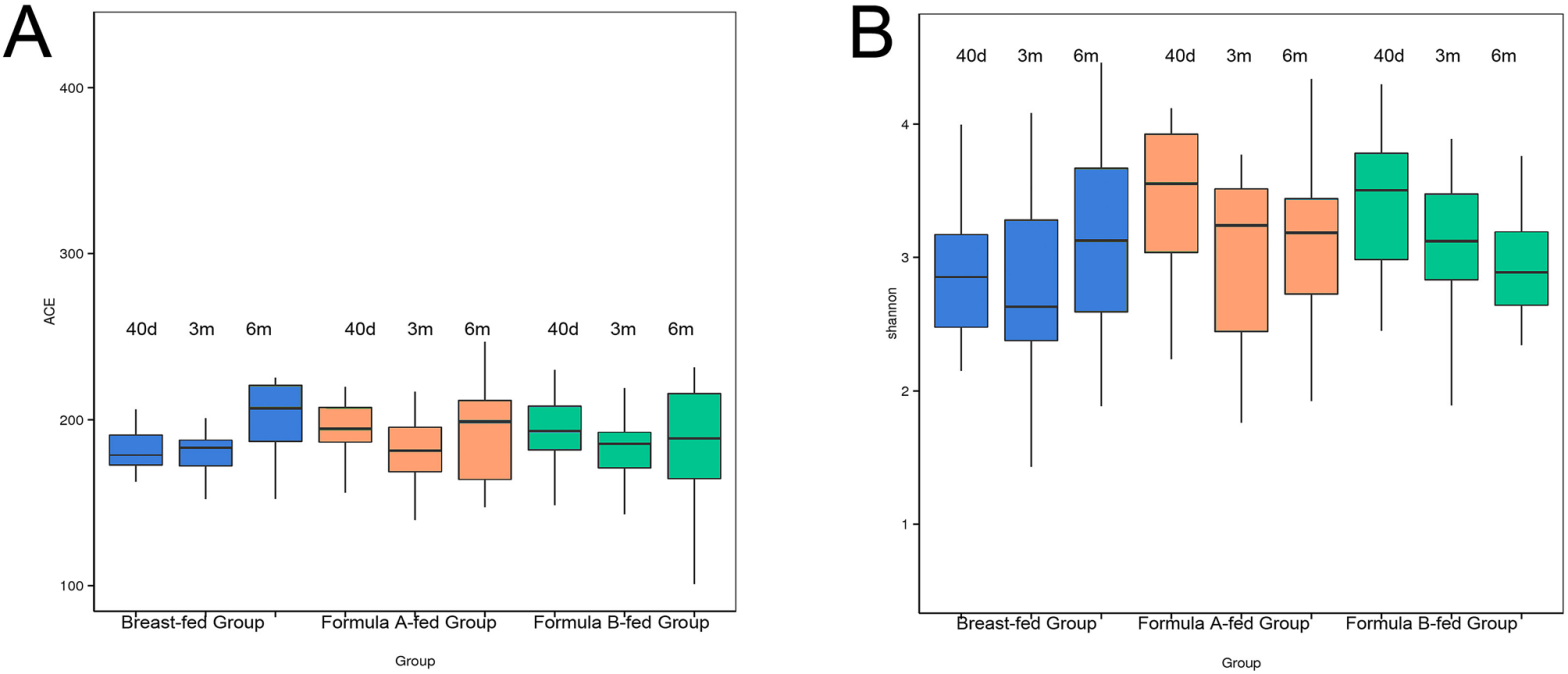
α diversity measurements using Ace index values indicated gut microbiota abundance (**A**), and Shannon index values indicated gut microbiota diversity (**B**). Y-axis represents the values of ACE (**A**) or Shannon index (**B**). Boxplots compare α diversity of gut microbiota in 40 days (40 d), 3 months (3 m), and 6 months (6 m) of age among breast-fed group, formula A-fed group, and formula B-fed group. Boxes show 25th to 75th percentiles and the median line, and whiskers indicate minimum to maximum values. Statistical significance was evaluated by Wilcoxon test, using p ≤ 0.05 as the measure of significance.

α diversity (within-sample diversity) measurements using Ace index values indicated gut microbiota abundance, and Shannon index values indicated gut microbiota diversity (Fig. 1; by Wilcoxon test). α diversity reported as Ace index indicated the bacterial communities remained unchanged in formula B-fed group (40 days versus 3 m p = 0.0766, 40 days versus 6 m p = 0.578, 3 m versus 6 m p = 0.2368). In breast-fed group, α diversity remained unchanged before 3 months of age (p = 0.6271), but increased significantly in 6 months of age (p = 0.0038). Shannon index values demonstrated an increasing trend of α diversity in breast-fed group and a decreasing trend in formula B-fed group over time, but the differences were not significant neither in breast-fed group (40 days versus 3 m p = 0.7627, 40 days versus 6 m p = 0.1483, 3 m versus 6 m p = 0.0827) nor in formula B-fed group (40 days versus 3 m p = 0.0276 > corrected p value 0.0167; 3 m versus 6 m p = 0.3557). While in formula A-fed group, Shannon index decreased significantly in 3 months (p = 0.0029) and then remained unchanged in 6 months of age (p = 0.6095). Shannon index showed a lower count in the breast-fed group compared to the formula-fed subjects in the 40-days old group (breast-fed versus formula A-fed p = 2e^−04^, versus formula B-fed p = 9e^−04^), but showed no significant differences with 3-months (breast-fed versus formula A-fed p = 0.2332, versus formula B-fed p = 0.1099) and 6-months old groups (breast-fed versus formula A-fed p = 0.9143, versus formula B-fed p = 0.2636).

### β diversity

**Figure 2:**
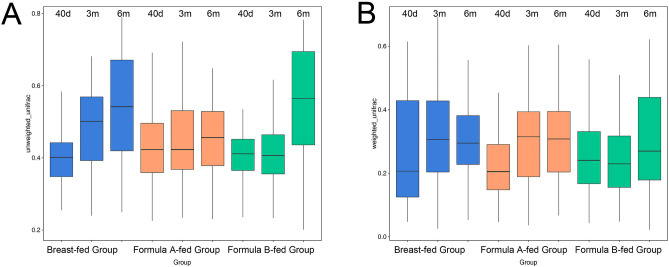
β diversity measurements by Unweighted UniFrac (**A**) and Weighted UniFrac (**B**). Boxplots compare β diversity of gut microbiota in 40 days (40 d), 3 months (3 m), and 6 months (6 m) of age among breast-fed group, formula A-fed group, and formula B-fed group. Boxes show 25th–75th percentiles and the median line, and whiskers indicate minimum to maximum values. Statistical significance was evaluated by Wilcoxon test, using p ≤ 0.05 as the measure of significance.

β diversity (between-sample diversity) was measured by Unweighted UniFrac and Weighted UniFrac (Fig. 2; by Wilcoxon test). β diversity of infant gut microbial community increased steadily during the first 6 months in breast-fed group (40 days versus 3 m p = 0, 40 days versus 6 m p = 0, 3 m versus 6 m p = 1e^−04^). In formula-fed babies, β diversity remained stable in 40 days and 3 months of age (40 days versus 3 m p = 0.5589 in formula-A group, p = 0.4525 in formula-B group), but increased significantly in 6 months of age (p = 0.0167 in formula-A group, p = 0 in formula-B group). In breast-fed group, β diversity was higher in 3 months of age than formula-fed groups (versus formula-A p = 0, versus formula-B p = 0). Compared with formula A-fed group, β diversity was lower in 40 days of age (p = 2e^−04^), and higher in 6 months of age (p = 0) in breast-fed and formula B-fed groups.

### Faecal microbial composition

**Figure 3:**
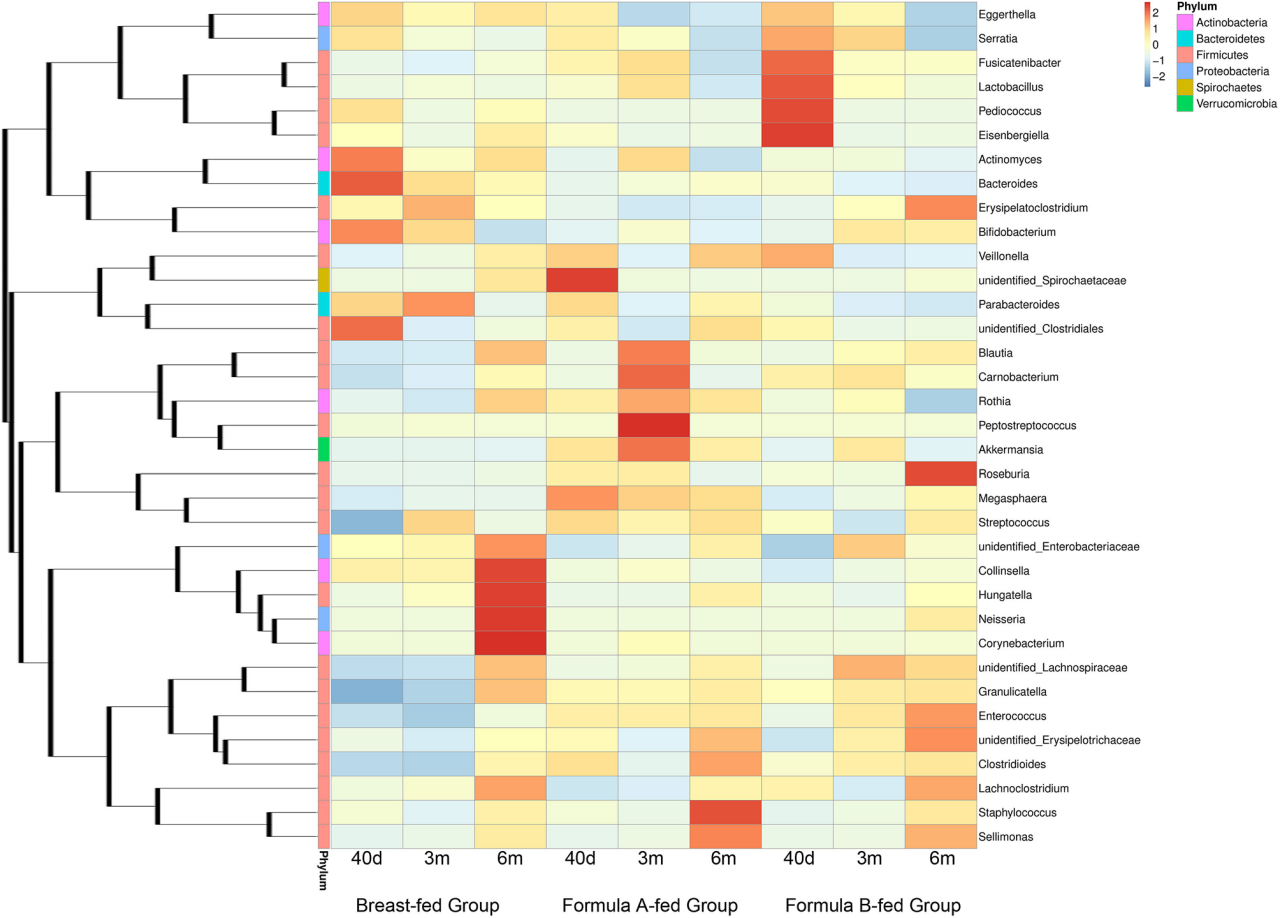
The OTUs heatmap at genus level. The relative abundance of OTUs was assessed across all samples, and OTUs were clustered in a heatmap according to their co-occurrence at genus level. Clustering was performed as a type of hierarchical clustering method to interpret the distance matrix using average linkage. The dendrogram provides the genus designation along the right Y-axis and the abundance relationship across all samples for each genus along the left Y-axis. The colour scale for the heatmap is shown in the upper right corner of the figure.

**Figure 4:**
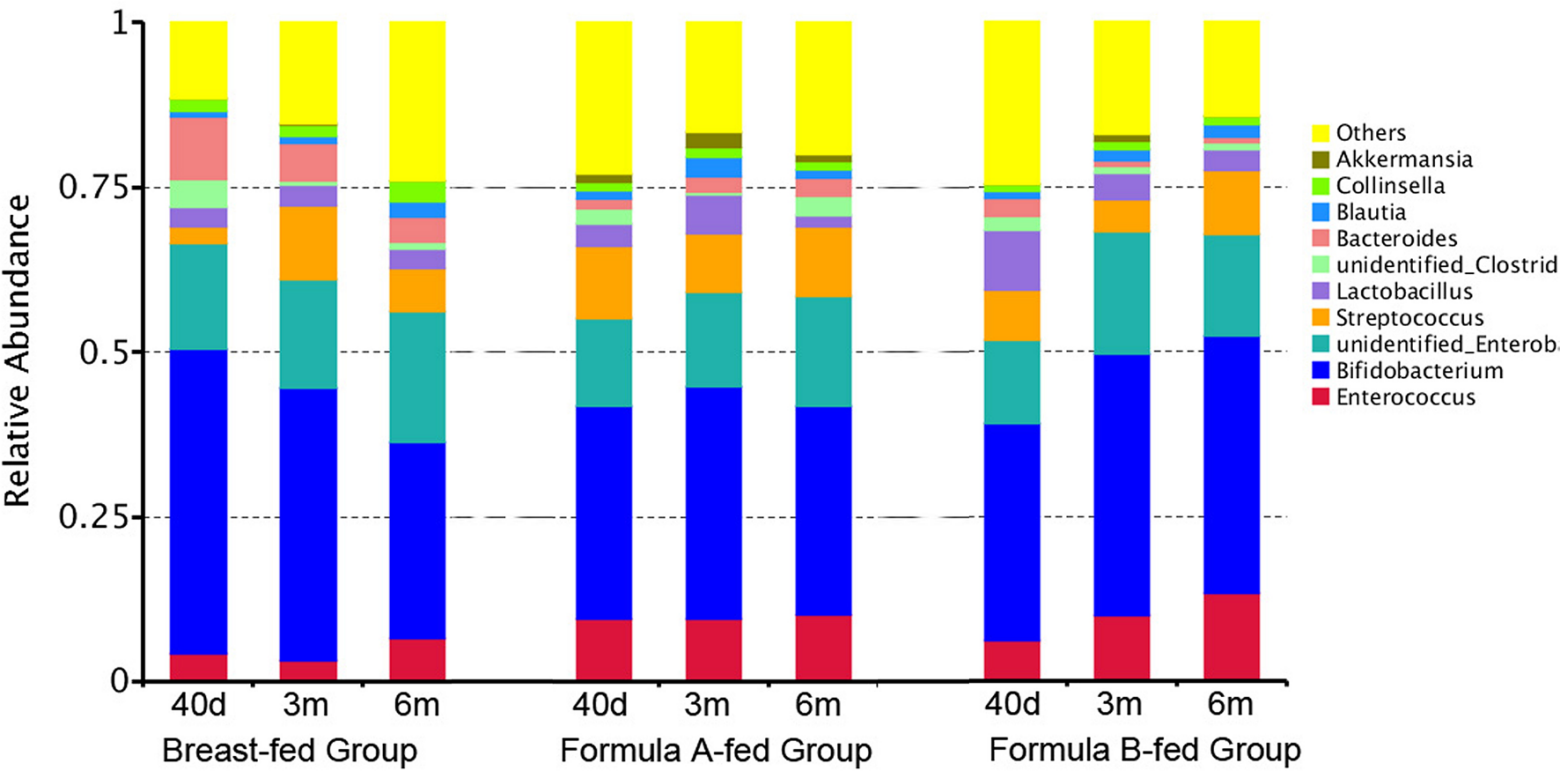
The ten most abundant bacteria of gut microbiota at genus level. Relative abundance estimates of the ten most dominant bacteria at genus level in 40 days, 3 months, and 6 months of age among breast-fed group, formula A-fed group, and formula B-fed group.

The relative abundance of operational taxonomic units (OTUs) was assessed across all samples, and OTUs were clustered in a heatmap according to their co-occurrence at genus level (Fig. 3). The *Bifidobacterium* represented the most predominant genus and *Enterobacteriaceae* the second in all groups at all time-points. *Bifidobacterium* accounted for 46.2%, 41.4% and 29.9% in breast-fed group, 32.2%, 35.3%, and 31.7% in formula-A group, and 33.0%, 39.8%, and 39.0% in formula-B group in 40 days, 3 months and 6 months of age, respectively. In formula-fed groups, the following were *Streptococcus* and *Enterococcus.* In breast-fed group, *Bacteroides* ranked third in 40 days (9.5%), but decreased as time went on to 5.9% in 3 m and 3.9% in 6 m. While *Enterococcus* and *Streptococcus* ranked third and fourth in 6 m in breast-fed group. After solid foods introduction, percentage of *Bacteroides* increased in formula A-fed group, from 2.3% in 3 m to 2.8% in 6 m, but kept almost the same in formula B-fed group from 0.9 to 0.8%. The 10 most abundant bacteria of gut microbiota at genus level were shown in Fig. 4.

### Succession of bacterial community and comparison among different groups

In our study, solid foods were introduced from 4 to 6 months of age, so they only affected the last time point in 6 m of age.

**Figure 5:**
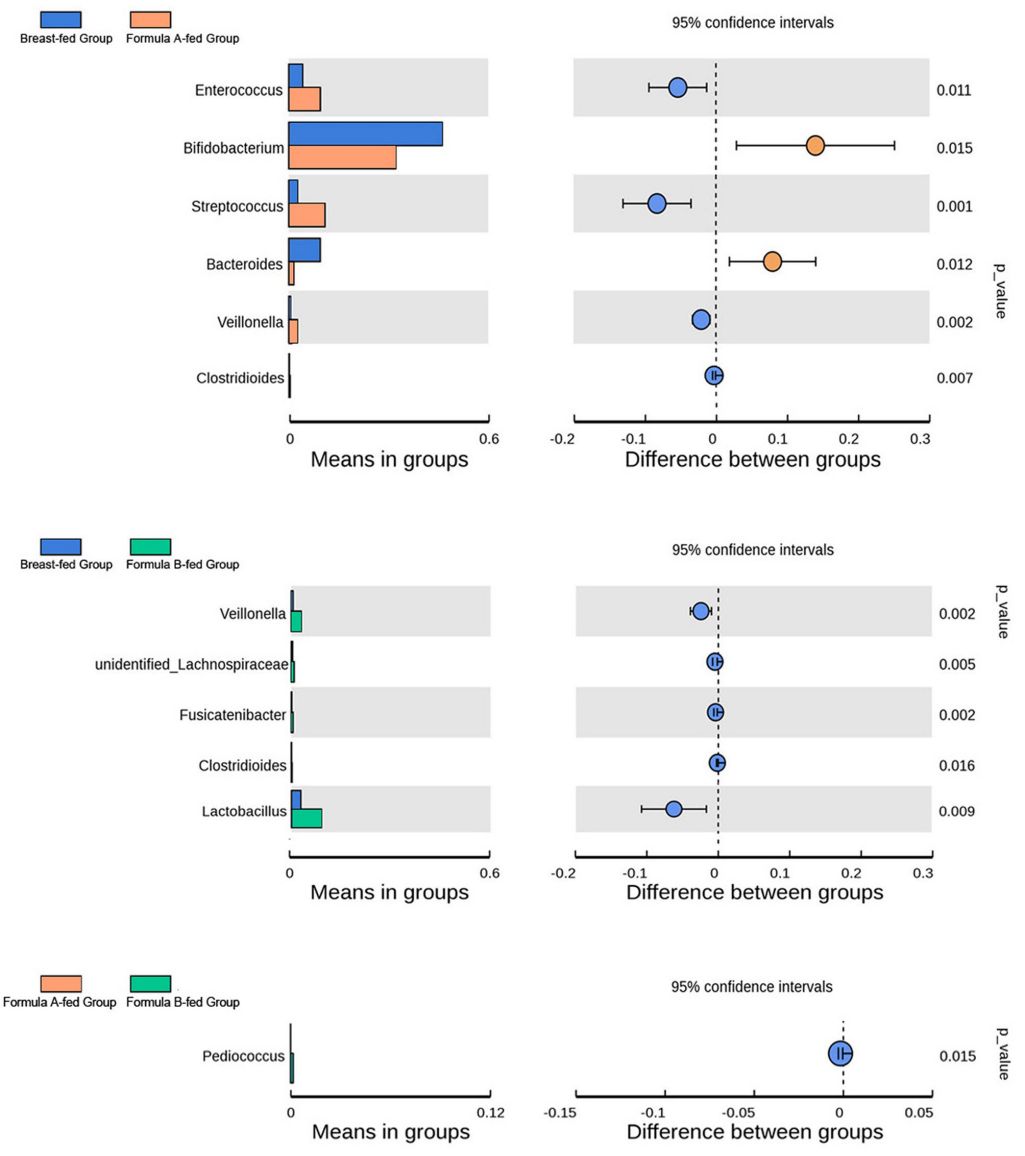
Gut microbiota comparison among different groups in 40 days of age. Bar graphs showing the relative abundance of the bacteria at genus level from breast-fed group, formula A-fed group, and formula B-fed group in 40 days of age. Variables were statistically tested by a two-tailed t test.

In 40 days of age, faecal *Veillonella* (p = 0.002, 0.002) and *Clostridioides* (p = 0.007, 0.016) were lower in breast-fed than those in formula A and formula B-fed groups. *Streptococcus* (p = 0.001) and *Enterococcus* (p = 0.011) copy numbers were significantly lower, while *Bacteroides* (p = 0.012) and *Bifidobacterium* (p = 0.015) were significantly higher in breast-fed group than they were in formula A-fed group. *Lachnospiraceae* (p = 0.005), *Fusicatenibacter* (p = 0.002) and *Lactobacillus* (p = 0.009) were lower in breast-fed group than those in formula B-fed group. *Pediococcus* (p = 0.015) was less in formula A-fed group than that in formula B-fed group (Fig. 5: variables were statistically tested by a two-tailed t test).

**Figure 6:**
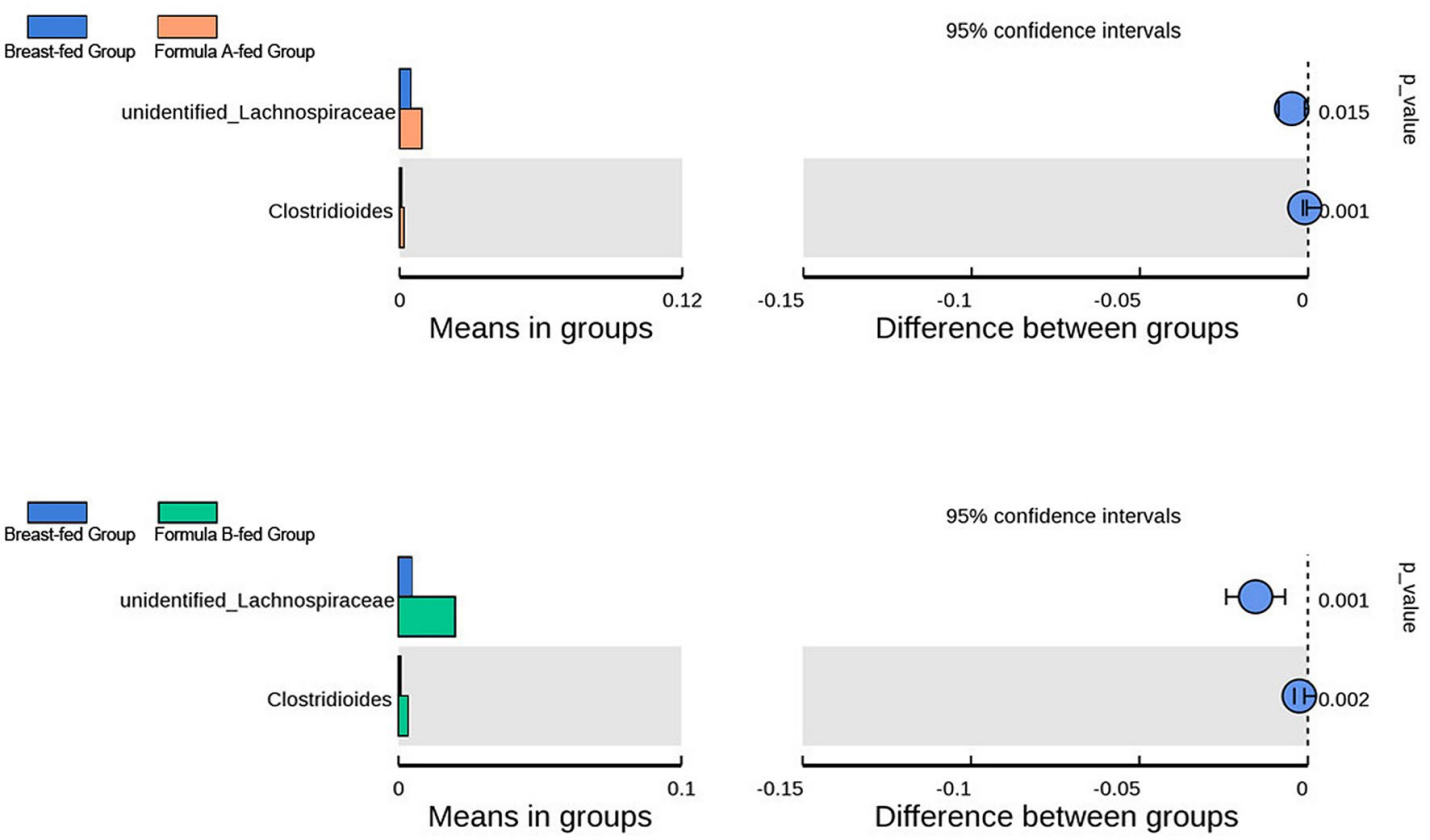
Gut microbiota comparison among different groups in 3 months of age. Bar graphs showing the relative abundance of the bacteria at genus level from breast-fed group, formula A-fed group, and formula B-fed group in 3 months of age. Variables were statistically tested by a two-tailed t test.

In 3 months of age, there were less *Lachnospiraceae* (p = 0.015, 0.001) and *Clostridioides* (p = 0.001, 0.002) in breast-fed group than that in formula A and formula B-fed groups. No differences were found between formula A and formula B-fed groups (Fig. 6: variables were statistically tested by a two-tailed t test).

In 6 months of age, no significant differences were seen among three groups.

### Correlation analysis between the differential bacteria at genus level and clinical parameters

In order to analyze the relationship between the gut microbiota and clinical parameters, we performed a correlation analysis (Kendall correlation coefficient for categorical variables, and Spearman for continuous variables) between the abundance of significantly altered bacteria at the genus level and parameters. In total, 13 covariates with known associations to gut microbiota development in infants were included in the analysis. Specifically, we analyzed maternal factors including age, height, weight, gestational weight gain, maternal prenatal antibiotics, and maternal postnatal antibiotics, as well as offspring factors such as mode of delivery (vaginal delivery, VD, or caesarean section delivery, CS), breastfeeding, antibiotics usage of infant, district (city or rural areas), vitamin D supplementation, household siblings and household furry pets.

The genera of *Bacteroides* and *Parabacteroides* were negatively correlated with the CS delivery. And the relative abundance of *Enterococcus* was positively correlated with antibiotics usage of infant (more details in Table 2).

**Table 2:** Significantly different correlations between clinical parameters and bacteria at genus level.

Parameters	Genera	Infant age	r	p value
Delivery mode of CS	*Bacteroides*	40 days	− 0.33227	0.010137
		6 m	− 0.33971	0.013744
	*Collinsella*	40 days	− 0.26796	0.040182
	*Parabacteroides*	40 days	− 0.34151	0.008116
	*Veillonella*	40 days	0.275663	0.034581
	*Clostridioides*	6 m	0.275826	0.047789
	*Akkermansia*	6 m	0.307399	0.026638
No maternal postnatal antibiotics usage	*Neisseria*	6 m	− 0.37933	0.005547
No antibiotics usage of infants	*Enterococcus*	3 m	− 0.27927	0.032189
	*Granulicatella*	6 m	0.274511	0.048905
	*Staphylococcus*	6 m	0.333008	0.048905
Maternal weight	*Terrisporobacter*	40d	− 0.29508	0.028741
	*Akkermansia*	6 m	− 0.33362	0.020489
	*Lachnoclostridium*	6 m	− 0.37552	0.008535
	*Carnobacterium*	6 m	0.309129	0.032523
Maternal weight gain	*Streptococcus*	40 days	− 0.27089	0.045458
Maternal height	*Bacteroides*	40 days	0.334113	0.012666
	*Lactococcus*	40 days	− 0.38638	0.003571
	*Terrisporobacter*	40 days	− 0.30384	0.024123
	*Subdoligranulum*	3 m	− 0.30344	0.02432
	*Dorea*	6 m	− 0.29074	0.044989
	*Streptococcus*	6 m	− 0.34579	0.01607
Maternal age	*Fusicatenibacter*	3 m	0.300977	0.025556
	*Enterococcus*	3 m	0.288757	0.032511
	*Lactobacillus*	3 m	0.386511	0.003559
	*Eubacterium*	6 m	− 0.36974	0.009696
	*Sellimonas*	6 m	− 0.3219	0.025669
No household siblings	*Hungatella*	40 days	0.264193	0.04318
	*Pediococcus*	40 days	0.272758	0.036611
	*Eisenbergiella*	6 m	0.306193	0.027269
District of rural areas	*Eubacterium*	3 m	− 0.27165	0.037411
	*Rothia*	3 m	0.282591	0.030114
	*Akkermansia*	6 m	0.40757	0.002709
No usage of vitamin D	*Eggerthella*	40 days	− 0.27819	0.032893
		6 m	0.351203	0.010683
	*Veillonella*	40 days	0.26331	0.043909
	*Actinomyces*	40 days	− 0.29616	0.022752
	*Lachnoclostridium*	40 days	− 0.2898	0.025987
	*Granulicatella*	3 m	0.272247	0.036978
	*Sellimonas*	6 m	0.316403	0.022301
	*Macellibacteroides*	6 m	0.314691	0.023076

The r values indicate correlation coefficient (Kendall for categorical variables, and Spearman for continuous variables), with minus values showing negative correlation, and positive values showing positive correlation.

## Discussion

We found that in breast-fed group, α diversity remained unchanged before 3 months of age, but increased significantly in 6 months of age. Previously studies have reported that faecal bacterial diversity increases with age, indicating a more complex microbial community over time^8,9^. Studies have shown that infants who are exclusively breast-fed have lower microbial diversity, compared with formula-fed babies whose gut microbiota is more diverse and similar to older children^10–12^. The difference of gut microbial diversity between breast-fed and formula-fed babies is also reported in animal research in tiger cubs^13^. We also found that among different groups, α diversity was lower in breast-fed group than formula-fed groups in 40 days of age. In adults, low gut microbial diversity has been linked to diseases in recent studies. In infants, breast milk may be the major determinant of a lower gut microbial diversity, because specific bacteria are selected for degrading particular oligosaccharides in breast milk. The predomination of infant-type *Bifidobacteria* during breastfeeding results in a low bacterial diversity, but it is beneficial for babies’ health. For example, the infant-type *Bifidobacteria* has a large impact on the maturation of the immune system, which may help reduce the incidence of infections in children. However, some diseases have been associated with a reduced microbial diversity in early life, such as eczema and asthma, which have been linked to low microbial diversity in 1 week–4 months of age. But the low microbial diversity is not coupled to *Bifidobacterium* abundance in these studies, and no reports have shown negative impacts of breastfeeding on development of asthma or allergies. The causality of lower diversity to diseases remains to be identified. What’s more, research has suggested that an immature gut microbial community can be “repaired” by introduction of adult-like microbes increasing greatly during introduction of solid foods in 6 months of age, which is within the development window of opportunity. Findings in adults cannot be inferred to infants regarding the association of gut microbial diversity with diseases, since the microbial ecosystem and the immune system of infants are quite different from adults^4^.

*Bifidobacterium* represented the most predominant genus and *Enterobacteriaceae* the second in all groups at all time-points in our study. Previous study also indicates that all infants have significant levels of *Enterobacteriaceae* and *Bifidobacteriaceae* at family level in 2 months of age. The abundance of a single genus usually constitutes the most in family level evaluation. Roger et al. have indicated that *Bifidobacterium* accounts for 40–60% on average of the total faecal microbiota of a 2-week old new born^10^. In our study, in 40 days of age, *Bifidobacterium* accounted for 46.2% in breast-fed group, and 32.2–33.0% in formula-fed groups, which was precisely classified according to feeding types. *Bifidobacterium* is present in the first few months and decreases as age goes on to almost zero by 18 months old^14^. *Enterobacteriaceae* also decreases with time^7,8^. This is consistent with the European study of 531 infants, which indicates the decrease trend in *Bifidobacteriaceae* and *Enterobacteriaceae* species from 6 weeks of age until 4 weeks after solid foods introduction, regardless of differences in feeding patterns^15^. We found that in breast-fed group, *Bifidobacterium* decreased from 46.2% in 40 days to 41.4% in 3 months and 29.9% in 6 months of age. In formula-fed groups, after solid foods introduction, *Bifidobacterium* decreased from 32.2% in 3 months to 31.7% in 6 months of age in formula A group, but increased from 33.0 to 39.0% in formula B group, indicating that different formulas may have different effects on microbiota. In our study, solid foods were introduced from 4 to 6 months of age, so they affected only the last time point in 6 m. We found that in 40 days of age, *Bifidobacterium* and *Bacteroides* were significantly higher, while *Streptococcus* and *Enterococcus* copy numbers were significantly lower in breast-fed group than they were in formula A-fed group. *Lachnospiraceae* was lower in breast-fed group than that in formula B-fed group. *Veillonella* and *Clostridioides* were lower in breast-fed group than that in formula A and B-fed groups. In 3 months of age there were less *Lachnospiraceae* and *Clostridioides* in breast-fed group than formula-fed groups. Other differences of microbiota were shown in Figs. 5 and 6.

After birth, the most important determinant of infant gut microbial colonization is breastfeeding. Studies have shown that breastfeeding is associated with higher levels of *Bifidobacterium*^1,2,16^, which is consistent with our study. The genus *Bifidobacterium* possesses multiple benefits, such as modulation of the immune system, production of vitamins, remission of atopic dermatitis symptoms in infants and decrease in rotavirus infections and lactose intolerance in children and adults^10,17^. *Bifidobacteria* is reported to be associated with diminished risk of allergic diseases^18^ and excessive weight gain^19^. Higher level of *Bifidobacteria* also indicates better immune responses to vaccines^20^.

*Bacteroides* is among several beneficial bacteria in the earlier neonatal phase, which has important and specific functions in the development of mucosal immune system^6^. The early activation of mucosal immune system may provide human body lifelong protection from health disorders^6^. *Bacteroides* is also linked with increased diversity and faster maturation of gut^2^. Koenig has studied 1 baby for 2.5 years after its birth and found that *Bacteroides* genus is absent before the introduction of solid foods^21^. However, Yassour M. et al. have reported that many infants present a significant *Bacteroides* species in the first 6 months, before the introduction of solid foods, in a longitudinal study of 39 children in their first 3 years of life^14^. We also found that there was *Bacteroides* in the first 6 months of life in all groups. *Bacteroides* was significantly higher in breast-fed infants, ranking third in 40 days (0.095) in breast-fed group, but decreased as time went on to 0.059 in 3 m and 0.039 in 6 m.

Besides *Bacteroides*, other health promoting bacteria like *Clostridia* has been reported to be vital to provide mucosal barrier homeostasis during the neonatal period, which is necessary in the immature intestine^6^. Formula-fed infants tend to have a more diverse microbial community with increased *Clostridia* species^9,12^, which is in accordance with our finding. We also found *Veillonella* was lower in breast-fed infants than formula-fed ones. Although there is an analysis indicating that *Veillonella* has been associated with a lower incidence of asthma, it has not taken feeding patterns into consideration^22^. So more data are needed to clarify the specific roles of certain bacteria with regard to feeding types.

Studies have shown that breast milk keeps the gut in a condition with a lower abundance of *Veillonellaceae*, *Enterococcaceae, Streptococcaceae*^9,11,23^ and *Lachnospiraceae*^7^, which is consistent with our results. Some researchers have indicated that higher level of *Streptococcus* sp. is seen in patients suffered from type 1 diabetes^2^. There may be other negative effects of these bacteria, but we still know little about them.

The subsequent big change in diet is the introduction of solid foods in 4–6 months of age, which is largely associated with changes in infant gut microbiota. A case study has found an increase in Bacteroidetes at phylum level after solid foods are introduced^21^. They have indicated that *Bacteroidetes* is specialized in the decomposition of complex plant polysaccharides^21^, and it is also associated with faster maturation of the intestinal microbial community^2^. In our study, after solid foods introduction, percentage of *Bacteroides* at genus level increased in formula A-fed group, from 0.023 to 0.028, but kept almost the same from 0.009 to 0.008 in formula B-fed group. While in breast-fed group, a decreased percentage of *Bacteroides* was found from 0.059 in 3 m to 0.039 in 6 m. The trends are different according to different feeding patterns. Pannaraj et al. believe that daily breastfeeding as a part of milk intake continues to affect the infant gut microbial composition, even after solid foods introduction^8^. But in our study, differences in gut microbiota between breast-fed group and formula-fed groups were not seen any more after solid foods were introduced. As for studies of gut microbiota, the taxonomic level of bacteria adopted in research may affect the results. We focused on microbiota mainly at genus level, resulting in certain discrepancies with some other articles at phylum or species level.

There were significant differences of microbiota between formula A-fed and formula B-fed groups in our study. We found that *Pediococcus* was less in formula A-fed group than that in formula B-fed group in 40 days. Many research articles have not taken the differences of formulas into consideration, especially retrospective studies. Even breast-fed group is mixed with formulas in some reports. So there must be some inaccuracies of their findings.

Except for feeding patterns, several factors are associated with the microbiota over the first year of life, which is a key period for the gut colonization, such as the mode of delivery, antibiotic exposure, geographical location, household siblings, and furry pets^2,9^. During the first days of life, the gut microbiota in infants born by vaginal delivery (VD) is similar to that in maternal vagina and intestinal tract, whereas in infants born by caesarean section delivery (CS) the gut microbiota shares characteristics with that of maternal skin. We noticed that the genera of *Bacteroides* and *Parabacteroides* were negatively correlated with CS. This was consistent with findings in many other studies, in which the difference of *Bacteroides* remains in 4 and 12 months of age^7,9^, and we also found the negative correlation of *Bacteroides* with CS existed not only in 40 days but also in 6 months of age. The increased morbidity reported extensively in infants born by CS is likely led by altered early gut colonization partially^24^. Accumulating data have indicated that antibiotic-mediated gut microbiota turbulence during the vital developmental window in early life period may lead to increased risk for chronic non-infectious diseases in later life^24^. There is a high detection rate of gut *Enterococcus* in antibiotic-treated infants in their early postnatal period among 26 infants born in a mean gestational age of 39 weeks^25^. We also found that the relative abundance of *Enterococcus* was positively correlated with antibiotics usage. The overgrowth of *Enterococcus* may be caused by antibiotic selection^25^.

In conclusion, by a larger cohort study than before, differences in gut microbiota among infants who were fed exclusively by breast milk or a single kind of formulas were obtained from this study, contributing further to our understanding of early gut microbial colonization, with more solid data than previous studies with mixed feeding patterns. Faecal diversity was lower in breast-fed infants than formula-fed ones in early life period, but increased significantly after solid foods introduction. A low diversity of the gut microbiota in early life appeared to characterize a healthy gut, if caused by breastfeeding, which was different from theories in adults. There were differences in bacterial composition in infants according to different feeding types, and even different formulas had different effects on microbiota, which we could not ignore in future research. This study presented initial data facilitating further research that will help us understand the importance of breastfeeding to gut microbiota in early life period.

## Limitations

Because the samples of exclusively breast-fed or formulas-fed babies were hard to collect by a single hospital, the subjects were recruited from two cities and four hospitals, all of which were members of North China Regional Union of Neonatologist, so that there might be selection bias in the enrolment of the study population. We did not analyse faecal metabolites which would be conducted in the future to better understand the function of gut microbiota. Our sampling did not include time points after 6 months of age, therefore our data did not provide information on trends in gut microbiota over time in relation to diet.

## Methods

We conducted a prospective study detecting gut microbiota in babies fed human milk exclusively or formulas exclusively for more than 4 months after birth.

### Study population

#### Inclusion criteria

(1) Healthy, full-term, new born babies. (2) Birth weight was ≥ 2.5 kg. (3) Babies were born between December 2016 to December 2017 in Peking Union Medical College Hospital, Inner Mongolia People’s Hospital, The Affiliated Hospital of Inner Mongolia Medical University, and Inner Mongolia Maternal and Child Health Hospital.

#### Group assignment

(1) Breast-fed group: Babies in the breast-fed group were fed breast milk exclusively for more than 4 months after birth. They were recruited in their regular follow-up in 40 days of age if they were fed breast milk exclusively at that time. (2) Formula-fed groups: Babies who have to be fed with formula due to mother’s disease or medicine and other objective reasons were potential subjects to our study. They were recruited before or right after birth. Parents chose formula A or B voluntarily after they signed the informed consents. Both formulas were market products with no reported adverse events. (1) Formula A-fed group: Babies were fed formula A (containing α lactalbumin and β casein) exclusively for more than 4 months after birth. (2) Formula B-fed group: Babies were fed formula B (containing α lactalbumin, β casein, as well as 1, 3-Olein-2-Palmitin) exclusively for more than 4 months after birth.

#### Exclusion criteria

(1) Gestational age < 37 weeks. (2) Birth weight was less than 2.5 kg. (3) Babies suffered from a serious disease such as heart failure, metabolic diseases, or congenital intestinal malformations. (4) Babies from breast-fed group could not be fed breast milk exclusively for 4 months for any reason. (5) Babies from formula A and B fed groups changed formula before 4 months for any reason.

### Study design

All infants were evaluated in 40 days, 3 months and 6 months of age. Clinical data and faecal samples were collected at each time point. Similar solid foods like infant cereals, purees and smashed fleshes were introduced to infants aged 4–6 months. The type and supplement order of solid foods were following the feeding guide of babies by the Chinese Nutrition Society in 2015.

#### Clinical data

Clinical data were collected including mothers’ conditions, such as combined diseases, antibiotics usage, age, height, weight and weight gain during pregnancy; and babies’ conditions, including mode of delivery, gestational age, gender, weight, length, head circumference, antibiotics usage, household siblings, pets, district, vitamin D supplementation, defecating frequency, stool property, and infections.

#### Samples collection

Faecal samples were collected from all infants at 40 days, 3 months and 6 months from birth. All the samples were kept in a sterile container, immediately stored in refrigerator at − 70 °C and sent to Beijing for testing collectively.

#### DNA extraction and sequencing

(1) Extraction of Genome DNA. Total genome DNA from samples was extracted using CTAB/SDS method. DNA concentration and purity was monitored on 1% agarose gels. According to the concentration, DNA was diluted to 1 ng/μL using sterile water. (2) Amplicon Generation. 16S ribosomal RNA (rRNA) genes of V4 region were amplified used specific primer (515F-806R) with the barcode. All PCR reactions were carried out with Phusion High-Fidelity PCR Master Mix (NEW ENGLAND BIOLABS). (3) PCR Products Quantification and Qualification. Mix same volume of 1X loading buffer (contained SYB green) with PCR products and operate electrophoresis on 2% agarose gel for detection. Samples with bright main strip between 400 and 450 bp were chosen for further experiments. (4) PCR Products Mixing and Purification. PCR products were mixed in equidensity ratios. Then, mixture PCR products were purified with Qiagen Gel Extraction Kit (QIAGEN, GERMANY). (5) Library Preparation and Sequencing. Sequencing libraries were generated using TruSeq DNA PCR-Free Sample Preparation Kit (ILLUMINA, USA) following manufacturer's recommendations and index codes were added. The library quality was assessed on the Qubit 2.0 Fluorometer (THERMO SCIENTIFIC) and Agilent Bioanalyzer 2100 system. At last, the library was sequenced on an Illumina MiSeq platform.

#### 16S rRNA gene sequence analysis

Paired-end reads was assigned to samples based on their unique barcode and truncated by cutting off the barcode and primer sequence. Quality filtering on the raw tags were performed under specific filtering conditions to obtain the high-quality clean tags^26^ according to the QIIME (V1.7.0, https://qiime.org/index.html)^27^ quality controlled process. A total of 20,383,186 reads (median 84,737 reads per sample) were obtained from 16S rRNA gene sequencing. Sequences analysis was performed by Uparse software v7.0.1001^28^. Sequences with ≥ 97% similarity were assigned to the same OTUs. Representative sequence for each OTU was screened for further annotation. For each representative sequence, the SILVA Database^29^ was used based on RDP classifier version 2.2^30^ algorithm to annotate taxonomic information. We compared differences in α diversity using Faith’s phylogenetic diversity. β diversity was evaluated by Principal Coordinate Analysis (PCoA) and PERMANOVA statistics on Unweighted and Weighted UniFrac distances.

### Statistical analysis

All statistical analyses were performed using IBM SPSS version 20.0 (IBM CO., ARMONK, NY, USA). Categorical variables were presented as proportions (percentages), and continuous variables were presented as (means ± standard deviation) or median (interquartile range). Normally distributed variables were statistically tested by a two-tailed t test for two independent groups or a one-way analysis of variance (ANOVA) for multiple independent groups. Nonnormal distributed variables were tested by Kruskal Wallis test. Inter-group differences were evaluated by χ^2^ test for categorical variables. Correlation analyses were performed by Kendall test for categorical variables, and Spearman test for continuous variables. A standard P value ≤ 0.05 was considered significant. A corrected P value ≤ 0.0167 was thought significant for multiple comparisons among three groups.

### Ethical approval and informed consent

Ethical approval was granted by the Ethics Institutional Review Board of Peking Union Medical College Hospital (protocol identifying number: HS-1148) on September 27, 2016. Informed consents were obtained from parents of the eligible infants. The study is in accordance with the ethical standards of the Declaration of Helsinki.

## Data Availability

All data and reagents could be made available from the corresponding authors upon request. Data including 16S rRNA gene data and metadata has been made available via the SRA database. The SRA accession number is SRP262038 (PRJNA633365).
